# Fluor Spar

**Published:** 1842-10

**Authors:** 


// THE WESTERN JOURNAL.

Vol. VI.—No. IV.

LOUISVILLE, OCTOBER 1, 1842

### FLUOR SPAR.

Dr. E. R. Roe, of Shawneetown, Illinois, wishes to inform scientific gentlemen through our journal that he can supply orders for *Fluate of Lime*, addressed to Drs. Roe & Badger at the place of his residence, if properly accompanied. He writes us that, since leaving Louisville last spring, he has received requests from various gentlemen to send them specimens of this mineral. He is prepared to furnish specimens to as many as may desire them. The mines are within about 20 miles of Shawneetown, in a wild, unsettled region of country. He says he has "spent some time and much pains in collecting specimens beautifully crystallized, and tinged with every shade of lilac and pink, up to pure transparency." Dr. Roe is a gentleman of science and industry, and may be depended upon for the faithful execution of all that he proposes. Y.

